# How to assess applicability and methodological quality of comparative studies of operative interventions in orthopedic trauma surgery

**DOI:** 10.1007/s00068-022-02031-9

**Published:** 2022-07-09

**Authors:** Kim Luijken, Bryan J. M. van de Wall, Lotty Hooft, Luke P. H. Leenen, R. Marijn Houwert, Rolf H. H. Groenwold, on behalf of the NEXT Study Group

**Affiliations:** 1 Department of Clinical Epidemiology, Leiden University Medical Center, Leiden, The Netherlands; 2 Department of Orthopedic and Trauma Surgery, Cantonal Hospital of Lucerne, Lucerne, Switzerland; 3 Department of Health Sciences and Medicine, University of Lucerne, Lucerne, Switzerland; 4 Julius Center for Health Sciences and Primary Care, University Medical Centre Utrecht, Utrecht University, Utrecht, The Netherlands; 5 Cochrane Netherlands, University Medical Centre Utrecht, Utrecht University, Utrecht, The Netherlands; 6 Department of Trauma Surgery, University Medical Center Utrecht, Utrecht, The Netherlands; 7 Department of Biomedical Data Sciences, Leiden University Medical Center, Leiden, The Netherlands

**Keywords:** Research applicability, Research methodology, Risk of bias, Systematic review, Emergency surgery

## Abstract

**Purpose:**

It is challenging to generate and subsequently implement high-quality evidence in surgical practice. A first step would be to grade the strengths and weaknesses of surgical evidence and appraise risk of bias and applicability. Here, we described items that are common to different risk-of-bias tools. We explained how these could be used to assess comparative operative intervention studies in orthopedic trauma surgery, and how these relate to applicability of results.

**Methods:**

We extracted information from the Cochrane risk-of-bias-2 (RoB-2) tool, Risk Of Bias In Non-randomised Studies—of Interventions tool (ROBINS-I), and Methodological Index for Non-Randomized Studies (MINORS) criteria and derived a concisely formulated set of items with signaling questions tailored to operative interventions in orthopedic trauma surgery.

**Results:**

The established set contained nine items: population, intervention, comparator, outcome, confounding, missing data and selection bias, intervention status, outcome assessment, and pre-specification of analysis. Each item can be assessed using signaling questions and was explained using good practice examples of operative intervention studies in orthopedic trauma surgery.

**Conclusion:**

The set of items will be useful to form a first judgment on studies, for example when including them in a systematic review. Existing risk of bias tools can be used for further evaluation of methodological quality. Additionally, the proposed set of items and signaling questions might be a helpful starting point for peer reviewers and clinical readers.

**Supplementary Information:**

The online version contains supplementary material available at 10.1007/s00068-022-02031-9.

## Background

It is challenging to generate and subsequently implement high-quality evidence in surgical practice [1]. In the field of orthopedic trauma surgery, it takes approximately 10 years from design to execution of an RCT [2]. What is more, Oberkofler and colleagues showed that results of surgical RCTs often do not convince the surgical community of their findings due to a perceived risk of bias [3]. This is a highly undesirable situation, because a lot of effort, time, public money, and patient participation is spent on research with negligible impact on surgical care [4].

To what extent a study can inform surgeons and patients depends on its applicability and (methodological) quality. Appraising the methodological quality of a study—especially the assessment of bias (internal validity)—and judging the applicability (external validity or generalizability) of study results to clinical practice remains challenging in the field of surgical research. This is reinforced by the fact that systematic reviews of operative interventions increasingly include both randomized controlled trials (RCTs) and observational studies [5, 6], adding to the complexity of the assessment.

Many comprehensive risk-of-bias tools are available to assess the methodological quality of studies of interventions [7–10], such as the Cochrane risk-of-bias (RoB 2) tool for randomized trials [11] and the Risk Of Bias In Non-randomised Studies-of Interventions (ROBINS-I) tool [12]. However, these tools focus on internal validity (risk of bias) aspects of a study and do not simultaneously evaluate clinical applicability of the results. The tools were often developed with a focus on studies of pharmacological interventions and may, therefore, not be ideally suited for studies of operative interventions. Additionally, it is convenient to assess both randomized and non-randomized studies using a single list of items.

Here, we describe items that are common to different risk-of-bias tools and formulated signaling questions that are tailored to operative interventions in orthopedic trauma surgery. We explain how these could be used to assess comparative operative intervention studies in orthopedic trauma surgery and how these relate to applicability of results. Selection of the items and their relation to existing risk-of-bias tools is described in Supplementary Material 1. We take the perspective of a researcher who performs a systematic review and wants to make a first judgment on the applicability and methodological quality of included studies. Relevance of the items for editors, peer reviewers, researchers, and clinical readers will be addressed in the discussion section. An illustration of how the proposed items can be used for assessment of applicability and methodological quality of randomized and non-randomized comparative studies into effects of operative interventions can be found in Supplementary Material 2, where the set of items was applied to re-assess studies that were included in two published systematic reviews of operative interventions for proximal humerus factures.

### Nine items to assess applicability and methodological quality

The established set contained nine items: population, intervention, comparator, outcome, confounding, missing data and selection bias, intervention status, outcome assessment, and pre-specification of analysis (Table 1). This set can be split in two subsets, representing applicability (first four items) and methodological quality (remaining five items). We discuss each item using examples from orthopedic trauma surgery literature.

**Table 1:** Overview of items and overarching questions of the established set

Applicability		
Item	Overarching question	Explanation
Population	Is the patient population included in the study representative of the patient population defined in the PICO of the systematic review?	Patients included in the study are ideally representative of patients that would be typically encountered in the clinical practice setting for which the PICO is defined
Intervention	Is the investigated intervention representative of the intervention defined in the PICO of the systematic review?	The studied operative intervention is ideally performed similar to the procedure that would be typically performed in the clinical practice setting for which the PICO is defined
Comparator	Is the comparator intervention representative of the comparator defined in the PICO of the systematic review?	The comparator intervention is ideally performed similar to the procedure that would be typically performed in the clinical practice setting for which the PICO is defined
Outcome	Is the outcome representative of the outcome defined in the PICO of the systematic review?	The outcome should be relevant to patients typically encountered in the clinical practice setting for which the PICO is defined and should be measured using an appropriate procedure at the appropriate time

Methodology		
Item	Overarching question	Explanation
Confounding	Is there comparability of intervention groups, or are appropriate methods applied to correct for incomparability?	An important assumption needed to make causal claims about effects of operative interventions is comparability of intervention groups. This can be established through appropriate randomization (in randomized studies) or adjustment for confounding (in observational studies)
Missing data and selection bias	Were the patients included in the analysis representative of all patients included in the study and was the impact of missing data negligible?	Selection of patients based on missing values or phenomena that occurred after inclusion into the study can introduce bias in the effect of the operative intervention
Intervention status	Was the intervention status correctly classified?	To infer the effect of an operative intervention it should be clear how crossovers and deviations from planned interventions are dealt with in the analysis
Outcome assessment	Was the outcome correctly measured?	The study outcome should be measured such that it does not influence the estimated effect by (dis)favoring the outcome of one of the intervention groups
Pre-specification of analysis	Were analyses prespecified and did the study adhere to the specified analysis plan?	Specifying analyses prior to analyzing the data prevents researchers from (often unintentionally) trying many approaches to fit the data, defining the research question/hypothesis only after observing the result, and selectively reporting the findings that yield the desired result

Supplementary Material 1 describes the signaling questions for each item

### Items of applicability of the evidence

The first four items represent the starting point of almost every clinical study, which is a clearly articulated research question. In a systematic review, the research question (see Box 1). In a systematic review, the research question determines which original studies should be included as well as the degree to which they can provide valuable evidence. If the research question of an original study does not match the research question of the systematic review, evidence can be indirect at most. The well-known PICO acronym can be a helpful structure when defining a research question about the possible effect of an operative intervention [13].

As an example, consider the following PICO for a systematic review: what is the effect of plate osteosynthesis (minimally invasive or open reduction and internal fixation) followed by 6 weeks non-weight-bearing functional treatment on functional outcomes measured using a validated functional score for the shoulder 1 year after the intervention compared to initiation of conservative intervention, consisting of 6 weeks pain-guided movement, no weight bearing, and a sling if necessary, in patients with a closed, displaced, proximal humerus fractures older than 18 years (Supplemental Material 2).

#### Population

The population defined in a research question ideally matches the patient population typically encountered in the clinical setting for which the PICO is defined. In orthopedic trauma surgery, elements that define the population are, e.g., the anatomical location of the fracture, the type of fracture (e.g., open/closed, simple/multifragmentary, or combination), and age group.

Fjalestad and colleagues [14] defined the relevant population as “*patients aged 60* + *years with a displaced, unstable three-or four-part proximal humerus fracture of OTA group 11-B2 or 11-C2 (displaced fracture of extra-articular or articular, bifocal type) without previous shoulder injuries*”. Because the population of interest was clearly reported (and its characteristics summarized in a table), the degree to which it matches to the population specified in the example PICO of the systematic review can easily be assessed.

#### Intervention

The studied operative intervention is ideally performed similar to the procedure that would be typically performed in the clinical practice setting for which the PICO is defined. To assess whether this is the case, a clear definition of the studied intervention should be given. In case of an operative intervention, this entails, e.g., specification of the osteosynthesis material, surgical approach and the type and duration of the post-operative treatment regime. In case of a conservative intervention, the duration and type of conservative intervention should be clearly reported.

For example, Fjalestad and colleagues [14] defined the studied intervention as follows: *“Patients allocated to surgery were operated on within 1 week of hospital admission. The goal of surgery was anatomic reduction of the fracture and fracture stabilization [using angular stable plate] to allow for early mobilization. After surgery, patients were immobilized in a modified Velpeau bandage until self-exercises and training instructed by a physical therapist were started on the third postoperative day.”* This was accompanied by a detailed account of the operative technique and the physiotherapy protocol, such that it was clear from the description what the intervention constituted. The intervention corresponds to the intervention defined in the example PICO of the systematic review, with the exception that the post-treatment regime was extended to include strengthening exercises after 6 weeks and a recommendation of physical therapy for at least 6 months.

Other relevant aspects of the intervention are whether study hospitals routinely perform the intervention, which help clarifying whether participating surgeons are experienced in conducting the investigated procedure. For instance, Fjalestad and colleagues indicated that: “*Three surgeons performed all operations and were trained in the surgical technique before performing surgery on study participants. Surgeons 1, 2, and 3 performed 18, five, and two operations, respectively. Surgery occurred during daytime hours*” [14]. Also, a learning curve (or the absence thereof) could be relevant. For example, Knobe et al. compared helical blade nailing of the femoral head versus locked plating and reported that [15]: “*[t]hree surgeons […] were proficient in the locked plating technique and three […] were proficient with helical blade nailing. Both implants had been used by the surgeons for more than 3 years, so they would have been beyond the learning curve and they had a comparable experience level for each implant*”.

#### Comparator

Similar to the studied intervention, the comparator intervention is ideally performed similar to the procedure that would be typically performed in the clinical practice setting for which the PICO is defined. To this end, the comparator intervention should be clearly defined, and the same considerations apply.

For example, Fjalestad and colleagues [14] defined the comparator intervention as follows: *“On admission to the hospital, patients were immobilized in a modified Velpeau bandage. All patients allocated to conservative treatment stayed in the hospital for at least 1 day and received the same instructions from the physiotherapist as patients allocated to surgery”*, accompanied by a description of an optional closed reduction procedure*.* The unambiguous reporting of the conservative treatment regime allowed for assessment of the applicability of the comparator arm with respect to the comparator specified in the example PICO of the systematic review. While the conservative intervention is roughly similar to the definition of the comparator intervention in the systematic review PICO, the optional closed reduction was not part of the systematic review PICO.

#### Outcome

The outcome should be relevant to patients typically encountered in the clinical practice setting for which the PICO is defined. Surrogate endpoints, such as laboratory or radiological findings, can turn out to be misleading substitutes for patient-important outcomes. Specification of a relevant study outcome consists of three parts: the outcome definition, the timepoint at which the outcome is assessed and the measurement procedure or instrument by which the outcome is assessed.

For example, Fjalestad and colleagues [14] defined the primary outcome as functional outcome at 1 year, indicating the outcome definition and timepoint at which it was assessed. The outcome measurement was the Constant score, which is a score ranging 0–100 measured by self-reported pain (max. 15 points), self-reported activities of daily-living (max. 20 points), range of motion (forward and lateral elevation, max. 10 points each, and external and internal rotation, max. 10 points each), and power (25 points) [16]. The unambiguous reporting of the outcome definition, timepoint and measurement procedure allowed for assessment of the applicability of the outcome with respect to the outcome specified in the example PICO of the systematic review.

### Items of methodology

Five items are key for assessing methodological quality of a study: confounding, missing data and selection bias, classification of intervention status, outcome assessment, and pre-specification of the statistical analysis. Each of the items will be discussed below.

#### Confounding

Comparability of intervention groups is essential for evaluation of effects of operative interventions and can be invoked by appropriate randomization (in randomized studies) or adjustment for confounding (in observational studies).

In randomized studies, a random allocation sequence and concealment of that allocation contribute to comparability of intervention groups, leading to comparability in observed (and unobserved) characteristics of study groups at baseline. An example of a clear description of the randomization procedure is given by Rangan and colleagues [17]: “*After obtaining informed consent and key baseline information, research associates randomly allocated patients to surgical or nonsurgical treatment using an independent remote randomization service (telephone or online access) provided by the York Trials Unit (University of York). Randomization was performed using a computer program with 1:1 allocation, stratifying by tuberosity involvement (yes or no) and using random block sizes of 4, 8, and 12.”* Based on this information, it can be assessed that the allocation sequence was random. Furthermore, inspection of baseline differences between intervention groups suggested no clinically relevant differences in observed characteristics. “*The baseline characteristics […] for randomized patients (N* = *250) and those providing [Oxford Shoulder Score] data at 2 years (n* = *215) were well balanced except for smoking status (there were more smokers in the nonsurgical group)*” [17].

Ideally, the allocation sequence is concealed at least until patients are enrolled in the study [18]. In case research associates or patients are aware which intervention the next enrolled patient will receive this might influence the decision to enroll (the patient) into the study and thus limit comparability of study groups. Hence, a detailed description of the allocation procedure is needed to assess the validity of the intervention allocation.

In observational studies, allocation of intervention is no random process, and intervention groups cannot be presumed to be comparable. Therefore, a key requirement for observational studies of operative interventions is that researchers argue convincingly that intervention groups are comparable or that they provide enough detail to assess whether important clinical characteristics are sufficiently controlled for in the statistical analysis of the study [19].

An example of the former is a study by Beks and colleagues, who compared the effect of rib fixation based on a clinical treatment algorithm on intensive care unit length of stay to nonoperative intervention for both patients with a flail chest and patients with multiple rib fractures [20]. They compared groups of patients with rib fractures admitted to hospitals that either operated most patients or mostly treated patients conservatively. Allocation of emergency patients to hospitals is to a certain extent a random process, based on availability and location of the accident. When different hospitals treat patients with similar symptoms with different interventions, this allows for a natural experiment by comparing outcomes across hospitals [21]. In this example, confounding due to severe incomparability of intervention groups was deemed unlikely by design. Additionally, Beks and colleagues adjusted for a number of confounders using propensity score matching.

Indeed, when intervention groups cannot be considered to be (fully) comparable by design, statistical adjustment for measured confounders can be considered. For example, Jenkinson and colleagues adjusted for variables that are considered to be confounders, because they are known risk factors of the outcome and they might have contributed to the indication for a particular intervention [22]: *“The factors considered to be the most important confounders also contributing to deep-infection risk were chosen for the propensity-score algorithm. These factors included patient age, sex, time delay to debridement, fracture grade (Gustilo-Anderson grade I, II, or IIIA), evidence of gross contamination, tibial compared with nontibial site, and ASA class (1 or 2 compared with 3 or higher). These factors were chosen, based on consensus among the investigators, as the factors most important for predicting later infection but also as those most divergent between the immediate and delayed-closure groups*”. Jenkinson and colleagues selected confounders based on background knowledge, in line with recommendations that specialist knowledge about the relation between covariates and the complex intervention and/or outcome is needed to identify a set of potential confounders.

A common misconception is that confounders can be identified based on statistical criteria. In fact, statistical criteria cannot identify nor discard covariates as being confounder variables [23–27]. Additionally, most statistical methods to adjust for confounding (including propensity score methods) can only adjust for measured confounding variables. After confounding adjustment, bias due to unmeasured confounding may still be present, e.g. because a confounder was measured inaccurately (or a continuous variable was dichotomized), or not measured at all [28]. A final note on confounders is that it is advisable not to interpret coefficients of confounding variables as causal effects or independent prognostic associations [29].

#### Missing data and selection bias

Data are often incomplete. In some circumstances, data can be missing without substantially affecting the results. When this is the case, a study report should clarify why missingness is thought to have no effect on the study outcome, as was done for example by Portinary and colleagues: “*To evaluate the impact of the emergency operations on postoperative functional status, the [activities of the daily living (ADL)] scores at the time of discharge were compared to the pre-admission ADL scores using the Chi-square test. Only patients for whom both pre-admission and postoperative ADL scores were available were included in this analysis. The subgroup analysis comparing patients with missing ADL score data with those where data was available showed no differences in terms of demographic and baseline characteristics […]. Therefore, participants without missing ADL score data were considered as a random sample of the study population. Therefore, missing data were considered to be completely at random and a complete case analysis was performed*” [30].

In many cases, however, excluding patients for whom information on some variables is missing can introduce bias, because the missingness is related to observed or unobserved characteristics of the patients [31–33]. Beks and colleagues assumed missingness in their study was at random and described how it was dealt with [20]: “*We applied multiple imputation (25 times) to impute missing values for ASA [2.1% (7/332)], TTSS [20% (67/332)], AIS head [0.6% (2/332)], pulmonary contusion [0.6% (2/332)], pH [9.0% (30/332)], and base excess [9.0% (30/332)]. Multiple imputation was performed using the mice() algorithm in R*”.

Studies should describe the reasons why data were missing (e.g., patient's death, retraction of informed consent) and discuss the assumed missing data mechanism. Otherwise, it is impossible to assess the potential impact of missing data and whether this was dealt with appropriately. The validity of performing a complete case analysis cannot be assessed from a study that merely states that patients with missing values were excluded from analysis. Pointing out that few cases were missing is not a valid justification of complete case analysis, since the proportion of missing data is not directly linked to the severity of the bias that is introduced by it [34].

Apart from variables having missing values, subjects can also be missing entirely in case they are not included in the study, which could lead to selection bias. However, if those included in the study are representative of the entire set of eligible subjects, the risk of this type of bias seems small. Klei and colleagues provided a clear description why patients included in the analyses seemed representative of all patients included in the study [35]: “*Among the 116 sternovertebral fracture patients, 43 patients were excluded from further analysis (1 military patient, 14 patients who died early after admission before fracture treatment, 14 patients with either isolated upper cervical spine or lower lumbar spine fractures, and 14 patients who were lost to follow-up). The remaining 73 patients were included for further analysis*”.

Sometimes, patients are excluded from analysis because they do not consent to participate in the study. A comparison between patients that consented and refused to partake in the study can be done to assess the possibility that selection bias is introduced, as is described by Rangan and colleagues [17]: “*Of the 563 eligible patients, 250 (44%) consented to take part in the trial […]. The mean age of the [consenting] participants was 66 years (range, 24–92 years), 192 (77%) were female, and 249 (99.6%) were white. These characteristics were similar to patients who refused consent (mean age, 68 years; 75% female).*”

#### Intervention status

The defined PICO specifies which operative interventions are compared—including their post-intervention regimens. However, deviations from these unambiguously defined yet potentially hypothetical situations can occur in clinical practice, both in the intervention and comparator arm.

One reason for such deviations is that the intervention status can be incorrectly registered in the data, referred to as ‘misclassification’. For instance, when data are retrieved from electronic health records, the procedure may be inaccurately registered or incorrectly extracted into the analytical dataset. A patient may falsely be recorded not to have received an operative intervention, while they actually had, or selection bias can be introduced in case of a comparison of operative interventions. However, in most cases, misclassification of surgical interventions seems unlikely.

Additionally, defining the intervention status of a patient in the final analysis is not straightforward when the patient was assigned to one intervention arm but actually received the opposite intervention (too). This is commonly referred to as a *cross-over*. Which intervention status patients should then be assigned to depends on the aim of the study, and in particular on the intervention effect of interest. For instance, an RCT by Van der Meijden and colleagues aimed to estimate an intention-to-treat effect and assessed patients in the intervention group that they were randomized to [36]. “*One patient (2%) in the plate group and six patients (10%) in the nailing group underwent intraoperative crossover to the other treatment group and were further analyzed as part of their original treatment group according to the intention-to-treat principle*”. Consequently, the result of the study no longer represents an effect of plate fixation versus intramedullary nailing on functional recovery. Rather, it represents an effect of plate fixation with optional revision using intramedullary nailing versus intramedullary nailing with optional revision using plate fixation on functional recovery. Although this interpretation is arguably less straightforward, it might be the effect of main interest in clinical practice.

Considerations regarding patients’ intervention status differ slightly between RCTs and observational studies. In RCTs, a cross-over commonly refers to a patient who was assigned to a particular intervention, but then received an alternative intervention, meaning that the patient received a single intervention. In observational studies, a cross-over commonly refers to a patient who received a particular intervention first and then received the alternative intervention, meaning that the patient received both interventions. Cross-over interventions in comparisons of operative versus non-operative interventions often pose a more challenging problem than crossovers between operative interventions.

Finally, including the post-operative treatment regime for determining a patient’s intervention status likely complicates matters considerably. Adherence to post-operative treatment is often less well documented and post-operative treatment options may be combined for some patients.

#### Outcome assessment

Ideally, the study outcome is measured in the same way in all study patients, notably irrespective of the intervention a patient received. This can be achieved by means of a valid and reliable procedure to measure the outcome [37]. For instance, the outcome ‘quality of life’ can be measured using a well-established questionnaire such as the EQ5D, as was done by Banierink and colleagues [38]: “*Quality of life was assessed with the EuroQol 5D (EQ-5D). The EQ-5D is a brief questionnaire that measures health-related quality of life based on five dimensions of health: mobility, self-care, usual activities, pain/discomfort and anxiety/depression *[17]*.*”

Functional outcomes are ideally measured using validated instruments, too, as was done by Ochen and colleagues [39]: “*Functional outcome was assessed at least 12 months following [the operative intervention], using the Dutch language version of the QuickDASH score. The QuickDASH is a validated and shortened version of the Disabilities of the Arm, Shoulder and Hand questionnaire (DASH)*”.

When the outcome is measured using a non-standardized measurement, outcome values may be less reliable. For instance, when forward or lateral elevation of the shoulder is measured by visual inspection rather than by use of a goniometer, values may be less reliable and inter-rater variability is likely increased. On top of that, an outcome assessor may (subconsciously) be affected by knowledge about the intervention that a patient received (i.e., when they are unblinded). These considerations apply to functional outcomes and self-reported outcomes, including patient reported outcome measures (PROMs), alike.

To prevent bias by unblinded outcome assessment, Nauth and colleagues designed their RCT anticipating the bias that could be introduced by differential unblinded outcome assessment of their primary outcome re-operation [40]: *“Surgeons and patients were not blinded. However, we did minimise the associated risk of bias with central and independent, although unblinded, radiographic adjudication of the primary endpoint”.* A committee adjudicated re-operations at the end of follow-up, where re-operation was defined as surgery to promote fracture healing, relieve pain, treat infection, or improve function within 24 months after the initial procedure (described in detail in the supplements of [40]).

#### Pre-specification of analysis

The credibility of results can be diminished by trying many approaches to fit the data and selectively reporting the results that yield the desired outcome. When the choice to perform a statistical test depends on patterns in the data, the expected number of false positives (i.e., type I error rate) is likely inflated [41]. Similarly, the type I error rate increases when more statistical tests are conducted on the same data set, thus performing multiple statistical tests without reporting all of them in the published manuscript prohibits readers from assessing the potential for false positive findings. Although such data dredging and cherry picking has harmful consequences, these practices may well be conducted unintentionally—especially when findings (in hindsight) are convincing and easy to explain. To overcome this problem, statistical data analysis should be prespecified as much as possible, e.g., by means of a statistical analysis plan that defines which analyses will be performed and the methods used to perform these analyses, including handling of missing data [42]. For RCTs, preregistration of the study protocol is considered the norm [43], but for observational studies, study protocols seem to be prespecified less often, although the urgency to do this is certainly recognized [44].

To further enhance transparency, protocols can be made publicly available to allow for assessment of protocol adherence. Protocols can be preregistered at, e.g., https://clinicaltrials.gov/ (for RCTs), https://www.isrctn.com/ (both RCTs and observational studies), https://osf.io/ (both RCTs and observational studies), and protocols for systematic reviews can be preregistered on https://www.crd.york.ac.uk/prospero/ or https://osf.io. Journals such as International Journal of Surgery Protocols or the British Medical Journal Open allow for publication of study protocols.

Good examples of publicly available study protocols are a trial by Smeeing and colleagues, who compared functional outcome twelve weeks after randomization to unprotected non-weight-bearing, protected weight-bearing, or unprotected weight-bearing as tolerated in patients who underwent surgical fixation of ankle fractures [45]. The protocol is available at https://www.trialregister.nl/, NTR3727 [46]. Taha and colleagues registered an observational pilot study to assess the feasibility of performing an RCT to study the effect of operative intervention of metacarpal fractures affecting the index to little finger(s) compared to non-operative intervention. The study is currently ongoing and is registered at ISRCTN (13,922,779).

Box 1: Well-definedness of research questions is crucial in studies of complex interventions
Studies investigating causal effects of interventions, both randomized and non-randomized, provide scientific evidence to inform medical decisions about those interventions. Ideally, a study indicates clearly which medical decision can be informed by the findings by unambiguously defining the research question, i.e., specifying the target population, the intervention strategies that are compared, and what outcome is considered and when.

In studies of pharmacological interventions, a research question could for instance be ‘what is the effect of taking drug A compared to taking drug B on a particular outcome in a specific population?’ Although this seems trivial, some parts of this question are not yet clearly defined. How is the drug administered (e.g., oral, or intravenous) and what dosages are compared? Other aspects, however, may be irrelevant, such as the hand with which a pill was taken or what shoes the individual was wearing when they took the drug. A research question should be sufficiently well defined in the sense that all relevant aspects are specified and thus should be addressed in the study design and analysis [60, 61].

Arguably, pharmacological interventions consist of less components than operative interventions and it is more straightforward to define them precisely. Studies of operative interventions go beyond a mere description of surgical techniques; other relevant aspects include the pre- and post-surgery treatment, experience of the surgeon and team, and more. On top of that, the operative intervention itself is tailored to a particular patient [62]. Hence, defining all relevant aspects in a research question demands considerable time and effort in studies of operative interventions.

For further reading on sufficiently well-defined research questions, we refer to [60] and [61].

## Discussion

We proposed a concise set of items, based on existing risk-of-bias tools, to perform an initial assessment of the applicability and methodological quality of randomized and non-randomized studies into effects of operative interventions in orthopedic trauma surgery. In terms of the IDEAL Framework [47–51], this set of items is intended to assess stage 3 (assessment) and stage 4 (long-term monitoring) studies. This assessment can be done as part of a systematic review to discard studies of low quality with relative ease and to separate out higher quality studies for further scrutiny of methodological quality using available assessment tools [11, 12, 52, 53]. In an accompanying study, the set of items was applied to re-assess studies that were included in two published systematic reviews of interventions for proximal humerus factures, providing an illustration of how the proposed items can be used for assessment of applicability and methodological quality of randomized and non-randomized studies into effects of operative interventions (Supplemental Material 2).

Guidance is increasingly developed on how to assess the credibility of research results and to grade the strengths and weaknesses of evidence provided in studies, particularly through Grading of Recommendations, Assessment, Development and Evaluations (GRADE) guidance [52, 53]. Compared to the comprehensive approach recommended by GRADE, the proposed set of nine items can be used as an initial assessment tool to identify high-quality studies that can be subject to further assessment.

As systematic reviews of operative procedures increasingly include both RCTs and observational studies [5, 6], it is convenient to evaluate both study types with the same set of items. Although RCTs have been described as being more internally valid than observational studies, as reflected in the traditional pyramid of evidence, it becomes increasingly apparent that randomization by design alone is insufficient as a surrogate for risk of bias [6, 21, 54], and revisions of the pyramid of evidence have been proposed [55]. Including both RCTs and observational studies in systematic reviews can be advantageous since they potentially provide complementary evidence on the effect of the studied operative intervention.

**Fig. 1:**
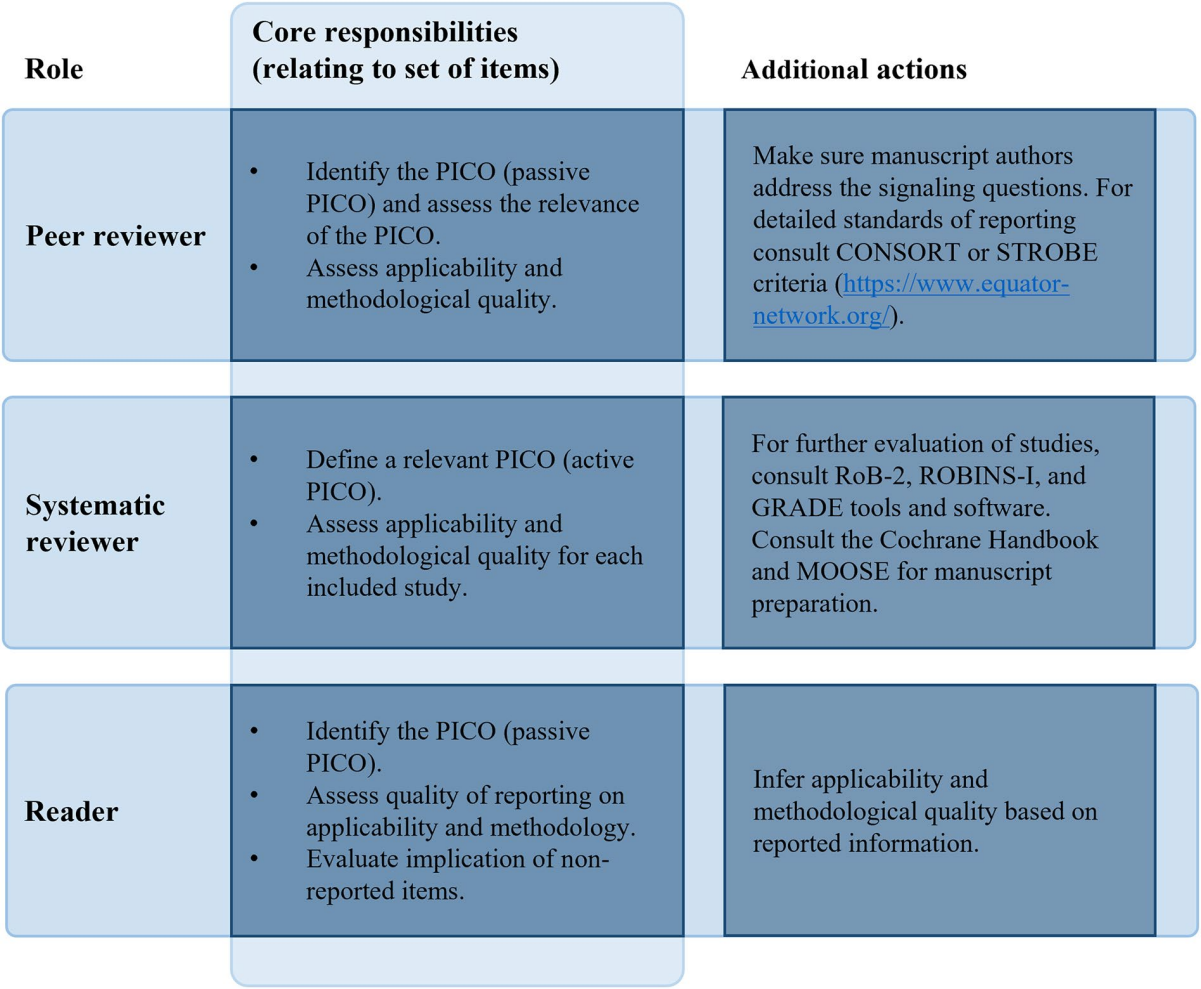
Schematic summary of how the reduced set of items can be used by peer reviewers, systematic reviewers, and other readers appraising studies of operative interventions. The contributed value of the set of items ranges from helpful instrument to mere starting point depending on the role of the assessor. When reporting on a study it can be useful to take into account that the study can be read from these perspectives

In the current study, we took the perspective of a systematic reviewer, who can use the set of items to appraise studies included in a systematic review and to determine which articles can be considered for a subsequent meta-analysis. However, the proposed set of items might be a helpful starting point also when taking on different roles (Fig. 1). The set of items can serve as a reference when peer reviewing an article or when informing medical decisions or policy. While the set of items is primarily derived for assessment of study reports (e.g., manuscript or published articles), it could be perceived as a starting point for researchers when they set up a study or when they report on their own research. However, given the many considerations involved in study conduct, it is advisable to consult other resources when working out a study design and analysis plan.

We intended to establish a set of assessment items that is easy to use with minimal loss of accuracy of the evaluation. The RoB-2 and ROBINS-I have been criticized for being time-consuming and requiring in-depth statistical knowledge, which would hinder their implementation in systematic reviews [56–58]. However, there is an evident trade-off in ease of use and rigor of the assessment. Uptake of rigorous assessment tools can be improved both by raising awareness and training in the use of available material [59] as well as by making the existing material more accessible. Our proposal is a first step towards bridging intelligibility and scrupulosity in assessment of studies of operative interventions. We encourage further development of an assessment instrument tailored to studies of operative interventions, in particular by bringing together surgical and methodological expertise. In light of such further developments, we point out that studies of operative interventions face a methodological challenge because most studies evaluate complex interventions and blinding is typically not feasible, but the current set of items does not explicitly address how to evaluate issues introduced by evaluations of complex interventions.

To conclude, the proposed set of items can be used for an initial assessment of the applicability and methodological quality of both randomized and non-randomized studies into effects of operative interventions. The items can discard studies of low quality with relative ease and separate out higher quality studies for further evaluation. We make a call to use this set not only when performing a systematic review and meta-analysis, but to use it as a reference also when peer reviewing an article, informing medical decisions or policy, or reporting on original research.

## Supplementary Information

Below is the link to the electronic supplementary material.

Supplementary file1 (PDF 406 KB)

Supplementary file2 (PDF 550 KB)

## Data Availability

Not applicable.
