# Supplementary material for: How to assess applicability and methodological quality of comparative studies of operative interventions in orthopedic trauma surgery
Source: Eur J Trauma Emerg Surg. 2022 Jul 9;48(6):4943–53. doi: 10.1007/s00068-022-02031-9 (PMC9712361; doi:10.1007/s00068-022-02031-9)
Supplement: Supplementary file 1 — Supplementary file1 (PDF 406 KB) [file 68_2022_2031_MOESM1_ESM.pdf]

Supplementary Material 1

This file is a supplement to 'How to assess applicability and methodological quality of comparative studies of operative interventions in orthopedic trauma surgery' by Kim Luijken, Bryan van de Wall, Lotty Hooft, Luke Leenen, Marijn Houwert and Rolf Groenwold and describes the signaling questions for the proposed set of items. Researchers can decide on the scoring options for each signaling question, such as yes/no/no information or yes/possibly yes/no/possibly no/no information. Where possible, we recommend documenting quotes that explicitly address a signaling question.

PICO of the systematic review

Population: ______________________________

Intervention: ______________________________

Comparator: ______________________________

Outcome: ______________________________

Applicability

Item	Question
Population	1. Is the patient population included in the study representative of the patient population defined in the PICO of the systematic review?
	1.1. Did inclusion criteria match the patient population specified in the PICO?
	1.2. Was a relevant subgroup of participants excluded?
Intervention	2. Is the investigated intervention representative of the intervention defined in the PICO of the systematic review?
	2.1. Was the investigated intervention similar to the intervention as defined in the PICO?
	2.2. Were the participating surgeons experienced in conducting the investigated procedure?
	2.3. Was the post-operative treatment regime in the intervention arm similar to the one defined in the PICO?
Comparator	3. Is the comparator intervention representative of the comparator defined in the PICO of the systematic review?
	3.1. Was the comparator similar to the comparator as defined in the PICO?
	3.2. Were the health care professionals experienced in conducting the comparator procedure?
	3.3. Was the post-intervention treatment regime in the comparator arm similar to the one defined in the PICO?
Outcome	4. Is the outcome representative of the outcome defined in the PICO of the systematic review?
	4.1. Was the outcome measurement similar to the outcome as defined in the PICO?
	4.2. Was the timing of the outcome described and similar to the specification in the PICO?

Methodology

Item	Question
Confounding	5. Is there comparability of intervention groups, or are appropriate methods applied to correct for incomparability?
	5.1. RCT: Was the allocation sequence random?
	5.2. RCT: Was the allocation sequence concealed until participants were enrolled and assigned to interventions?
	5.3. RCT: Did baseline differences between intervention groups suggest a problem with the randomization process?
	5.4. Obs: Is there potential for confounding of the effect of the intervention in this study?
	5.5. Obs: Did the authors use an appropriate analysis method that controlled for all the important confounders?
	5.6. Obs: If 5.5. = Y or PY, were confounders that were controlled for measured adequately?
Missing data and selection bias	6. Were the patients included in the analysis representative of all patients included in the study and was the impact of missing data negligible?
	6.1. Were outcome data available for all, or nearly all, participants?
	6.2. Obs: Were intervention data available for all, or nearly all, participants?
	6.3. Obs: Were confounder data available for all, or nearly all, participants?
	6.4. If 6.1./6.2./6.3. = N or PN: were convincing arguments given for complete case analysis or were methods applied to address missing data?
	6.5. Was selection of participants into the study (or into the analysis) based on variables measured after the start of the intervention?
	6.6. Do start of follow-up and start of intervention coincide for all, or nearly all, participants?
Intervention status	7. Was the intervention status correctly classified?
	7.1. Did the recorded intervention status correspond to the intervention actually received?
	7.2. Was there cross-over between interventions or non-adherence to the assigned intervention regimen that could have affected participants' outcomes?
	7.3. If 7.2. = Y or PY, was an appropriate analysis used to estimate the effect of starting and adhering to the intervention?
Outcome assessment	8. Was the outcome correctly measured?
	8.1. Was the outcome measurement a valid and reliable measurement of the outcome?
	8.2. Were outcome assessors aware of the intervention received by study participants?
	8.3. Were the methods of outcome assessment comparable across intervention groups?
Pre-specification of analysis	9. Were analyses prespecified and did the study adhere to the specified analysis plan?
	9.1. Was the analysis prespecified, e.g., in a protocol?
	9.2. Are the reported results likely to be a selection of results of multiple analyses?

Extracting items from existing risk-of-bias tools

To establish an easy-to-use set of items for assessment of applicability and methodological quality of studies of operative interventions, we extracted information from the RoB-2[1], ROBINS-I[2], and MINORS criteria[3] and derived a concisely formulated set of items tailored to operative interventions. It has been pointed out that the methodologically rigorous RoB-2 and ROBINS-I tools require a high level of statistical knowledge, making their implementation challenging and time consuming[4-6]. We aimed to summarize scoring items in such a way that the items were easy to use for assessment of articles of both RCTs and observational studies of operative interventions.

All signaling questions from the RoB-2, ROBINS-I, and MINORS were taken as a starting point. We identified signaling questions with overlapping topics. Based on their relevance to surgical studies, the overlapping set formed the initial key items. We then evaluated remaining signaling questions. Questions that were less relevant for studies of operative interventions, such as questions regarding time-varying exposures, were discarded and questions were reformulated to be more appropriate for a surgical context (Figure S1). The set of items was further improved by user experiences in an accompanying study that assessed the applicability and methodological quality of studies from two recent systematic reviews (Supplementary File 2).

The finally established set contained 4 items on applicability and 5 items of methodology (described in the Table above). Each item contained multiple signaling questions that help to arrive at an overarching judgement of the study quality regarding that topic, with some signaling questions specifically applicable only to RCT or observational studies. The proposed set aimed to summarize key information needed to assess the applicability and methodological quality of operative intervention studies, but the choice for items and signaling questions was surely arbitrary, and the set is opened to further elaborations and improvements.

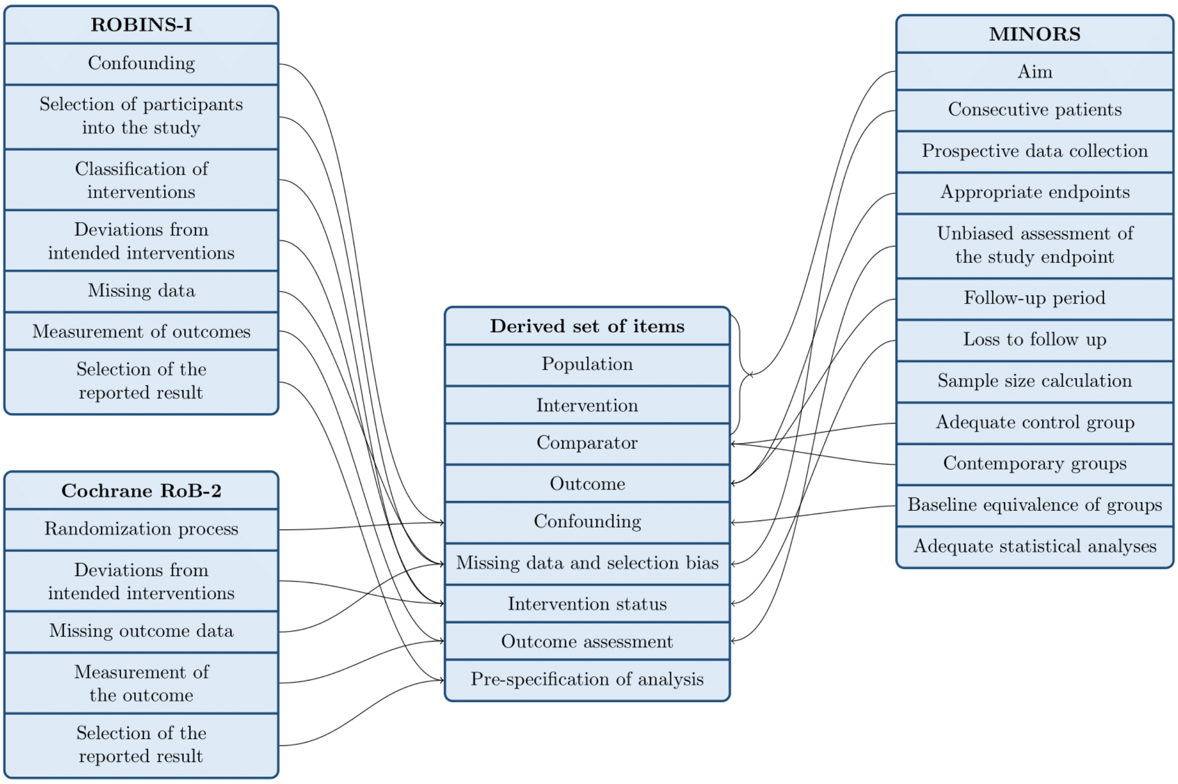

Figure S1. Flow diagram indicating which existing risk of bias tool the signaling questions in the reduced set were based on.

References

1. Sterne, J.A., et al., *RoB 2: a revised tool for assessing risk of bias in randomised trials.* bmj, 2019. **366**.
2. Sterne, J.A., et al., *ROBINS-I: a tool for assessing risk of bias in non-randomised studies of interventions.* bmj, 2016. **355**.
3. Slim, K., et al., *Methodological index for non-randomized studies (MINORS): development and validation of a new instrument.* ANZ journal of surgery, 2003. **73**(9): p. 712-716.
4. Jeyaraman, M.M., et al., *Methodologically rigorous risk of bias tools for nonrandomized studies had low reliability and high evaluator burden.* Journal of clinical epidemiology, 2020. **128**: p. 140-147.
5. Minozzi, S., et al., *The revised Cochrane risk of bias tool for randomized trials (RoB 2) showed low interrater reliability and challenges in its application.* Journal of clinical epidemiology, 2020. **126**: p. 37-44.
6. Minozzi, S., et al., *Risk of bias in nonrandomized studies of interventions showed low inter-rater reliability and challenges in its application.* Journal of clinical epidemiology, 2019. **112**: p. 28-35.
