# Supplementary material for: How to assess applicability and methodological quality of comparative studies of operative interventions in orthopedic trauma surgery
Source: Eur J Trauma Emerg Surg. 2022 Jul 9;48(6):4943–53. doi: 10.1007/s00068-022-02031-9 (PMC9712361; doi:10.1007/s00068-022-02031-9)
Supplement: Supplementary file 2 — Supplementary file2 (PDF 550 KB) [file 68_2022_2031_MOESM2_ESM.pdf]

Supplementary Material 2

This file is a supplement to 'How to assess applicability and methodological quality of comparative studies of operative interventions in orthopedic trauma surgery' by Kim Luijken, Bryan van de Wall, Lotty Hooft, Luke Leenen, Marijn Houwert and Rolf Groenwold and describes an illustration of how the proposed items can be used for assessment of applicability and methodological quality of randomized and non-randomized studies into effects of operative interventions. In this accompanying study, the set of items was applied to re-assess studies that were included in two published systematic reviews of interventions for proximal humerus factures.

Bryan J.M. van de Wall[1,2], Kim Luijken[3], Steven Ferree[4], Ingmar Rompen[1], Frank JP Beeres[1], Luke PH Leenen[5], R. Marijn Houwert[3,5], Rolf H.H. Groenwold[3,5], on behalf of the NEXT Study Group

[1] Department of Orthopedic and Trauma Surgery, Cantonal Hospital of Lucerne, Lucerne, Switzerland.

[2] Department of Health Sciences and Medicine, University of Lucerne, Lucerne, Switzerland.

[3] Department of Clinical Epidemiology, Leiden University Medical Center, Leiden, the Netherlands.

[4] Department of Surgery, Diakonessenhuis, Utrecht, the Netherlands.

[5] Department of Trauma Surgery, University Medical Center Utrecht, Utrecht, the Netherlands.

[6] Department of Biomedical Data Sciences, Leiden University Medical Center, Leiden, the Netherlands.

Abstract

Purpose: The aim of this study was to 1) Investigate the effects of broadening or narrowing down the research question of a systematic review on the applicability of included studies; 2) Describe methodological challenges in randomised clinical trials (RCT) and observational studies that were encountered in two systematic reviews of interventions for proximal humerus factures.

Methods: We assessed the applicability and methodological quality of surgical intervention studies (24 observational studies and 8 randomised trials) that were included in two recently performed systematic reviews. These reviews focused on operative versus operative and operative versus conservative treatment for proximal humerus fractures.

Results: Broadening definitions of individual components of a research question (population, intervention, comparator, outcome) was found to increase the applicability of studies included in the systematic reviews. Methodological issues encountered in both RCTs and observational studies were a lack of clear clinical outcome definitions and pre-specification of the design and analysis of a study in a published protocol. Additionally, observational studies showed a high risk of bias due to confounding, however, attempts to control for confounding were rarely performed.

Conclusion: The number of studies that can be included in a systematic review heavily depends on how specific the research question is articulated. Both RCTs and observational studies in orthopaedic trauma research face recurring, but solvable, methodological challenges including the lack of control for confounding and clear outcome definitions. Establishing and publishing a protocol for all studies, irrespective of design (RCTs and observational studies), might overcome these problems as it forces researchers to think about critical design aspects prior to conducting a study.

Introduction

Medical decisions are ideally based on state-of-the-art evidence that includes recent insights from scientific research. For example, decisions about surgical interventions in orthopedic trauma patients could be informed by systematic reviews summarizing the best available evidence from multiple studies. This requires an adequate assessment of both applicability and methodological quality of those studies, which can be a daunting task.

In a separate paper, we described a set of nine items for initial assessment of applicability and methodological quality of studies of surgical interventions that can be used to form a first judgment on studies that have been included in a systematic review.[1] The set of key items was informed by user experiences from the current study, in which we reappraised two previously conducted systematic reviews on the effect of operative treatment of proximal humerus fractures.[2, 3]

In what follows, we describe our experiences with the reappraisal regarding two aspects. Firstly, we describe the effects of broadening and narrowing down the research question on applicability of studies included in the previously conducted systematic reviews. Secondly, we investigate what methodological issues can be encountered in randomised clinical trials (RCT) and observational studies using the two systematic reviews as an example.

Methods

Two recently conducted systematic reviews on surgical treatment for proximal humerus fractures were included: one comparing conservative to operative treatment and the other comparing one operative technique (open reduction interval fixation with plate) to an alternative operative technique (minimally invasive plating).[2, 3] These studies were chosen because the clinical topic is the same, yet the nature of treatments being compared is different.

Definition PICO

Prior to assessment of applicability of all studies included in the two systematic reviews, two types of research questions were formulated in terms of population (P), intervention (I), comparator (C) and outcome (O): the research question as described in the original systematic reviews (which was defined relatively generically, or broadly) and a more specifically (or narrowly) defined research question. For each systematic review, these research questions are described in Table 1.

Part 1- Assessment of applicability of studies

Key information about study population, intervention, comparator and outcome definitions was extracted independently by two authors (BW, SF) from all studies included in the two systematic reviews. This information was used to assess the applicability of the studies regarding the previously described PICOs.

Table 1. Definition of the broad PICO and narrow PICO.

PICO	Population	Intervention	Comparator	Outcome (clinical)	Outcome (functional)
**Broad**	Proximal humerus fractures in patients older than 18 years	Operative treatment excluding external osteosynthesis	Conservative treatment	Non-union	Constant score at least one year after initialisation of treatments
**Narrow**	Closed, displaced, proximal humerus fractures in patients older than 18 years	Plate osteosynthesis (open or minimally invasive) followed by functional after treatment (6 weeks none weight bearing)	Sling as needed, pain guided shoulder movement, 6 weeks no weight bearing	Non-union defined as persisting pain in the fracture zone with no radiological signs of healing (callus, disappearing fractures lines) at 9 months follow-up	Functional scores measured one year after follow-up using validated shoulder scores
**Broad**	Proximal humerus fractures	Minimally invasive plate osteosynthesis	Open reduction internal fixation	Union (not further specified)	Functional scores (not further specified)
**Narrow**	Dislocated proximal humerus fractures Neer III and IV (no luxationfractures)	Minimally invasive (anterolateral or deltoid split) plate osteosynthesis using philosplate followed by functional after treatment (6 weeks none weight bearing)	Open reduction (deltopectoral approach) internal fixation using philosplate followed by functional after treatment (6 weeks none weight bearing)	Radiological union defined as bridging callus or fading of the fracture lines in three out of four cortices on conventional anteroposterior and lateral X-rays	Functional scores measured at 6-12 months using validated shoulder scores

* Broad: PICO as described in the included meta-analyses

~Narrow: PICO as composed by the authors of the present umbrella review

Part 2 – Assessment of methodological quality of studies

The same two authors independently scored the methodological quality of the included studies according to five methodological items: confounding, missing data and selection bias, intervention status, outcome assessment, and pre-specification of analysis. For the item "outcome assessment", the methodological quality was scored separately for functional outcomes and clinical outcomes described in the included studies. Clinical outcomes can be measured relatively objectively and are frequently based on events requiring (operative or medical) interventions, radiological, biochemical or microbiological outcome data in surgical research.[4] In the examples we used the clinical outcome union and non-union (see Table 1 for details). Functional outcomes in the present study were outcomes measured by validated scoring tools designed to measure function of the shoulder joint.

Data synthesis

All studies were scored using a set of 33 signaling questions (scoring options: Yes/Probably yes/No/Probably no/No information), based on which the item scores were obtained, as described in the tables at the end of this file. For each item, a study could score "no information", "poor", "moderate" or “good” applicability or methodological quality. Results were stratified by the original systematic review.

Results on methodology items were additionally stratified according to study design (RCT and observational studies).

Results

Characteristics of included studies

The systematic review on conservative versus operative treatment for proximal humerus fractures consisted of 6 RCTs and 16 observational studies. The review on open reduction internal fixation versus minimally invasive plating had 2 RCTs and 14 observational study. Six articles about retrospective observational studies included in the original systematic review eventually had to be excluded from the present analysis as these were written in Chinese.

Part 1- Applicability of studies

Figure 1 shows the applicability of studies included in the two systematic reviews using the PICO of the systematic review itself (broad PICO) and a more narrowly defined PICO composed by the authors of the present study (narrow PICO). In general, when the PICO is defined more narrowly, the applicability of results of individual studies reduces. The extent to which this phenomenon was observed differed between the reviews. It was predominantly found when narrowing down the definition of the study population component in the review on minimally invasive plating versus open reduction internal fixation and, also, for the intervention/comparator component in the review on conservative versus operative treatment.

Figure 1. Applicability of results of the 32 included studies using broad and narrow PICO definition in the two systematic reviews.

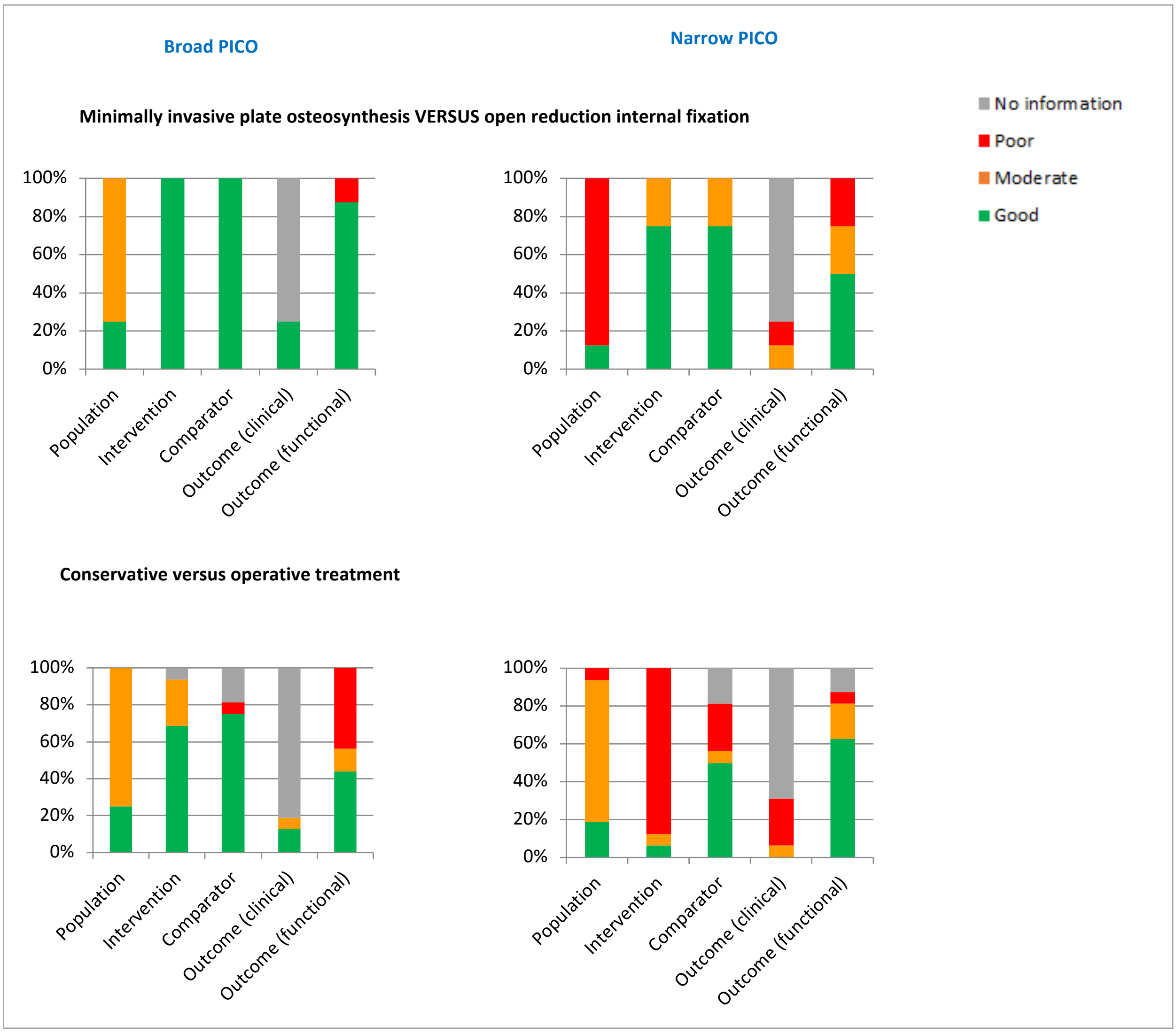

Part 2 - Methodological quality of studies

Figure 2 demonstrates the methodological quality of RCTs (n=8) and observational studies (n=24) included in the two systematic reviews on treatment for proximal humerus fractures. In this example, some notable differences between both study designs were observed.

Figure 2. Methodological quality according five domains for all studies included in the two systematic reviews, stratified for study design (observational studies versus randomised clinical trials).

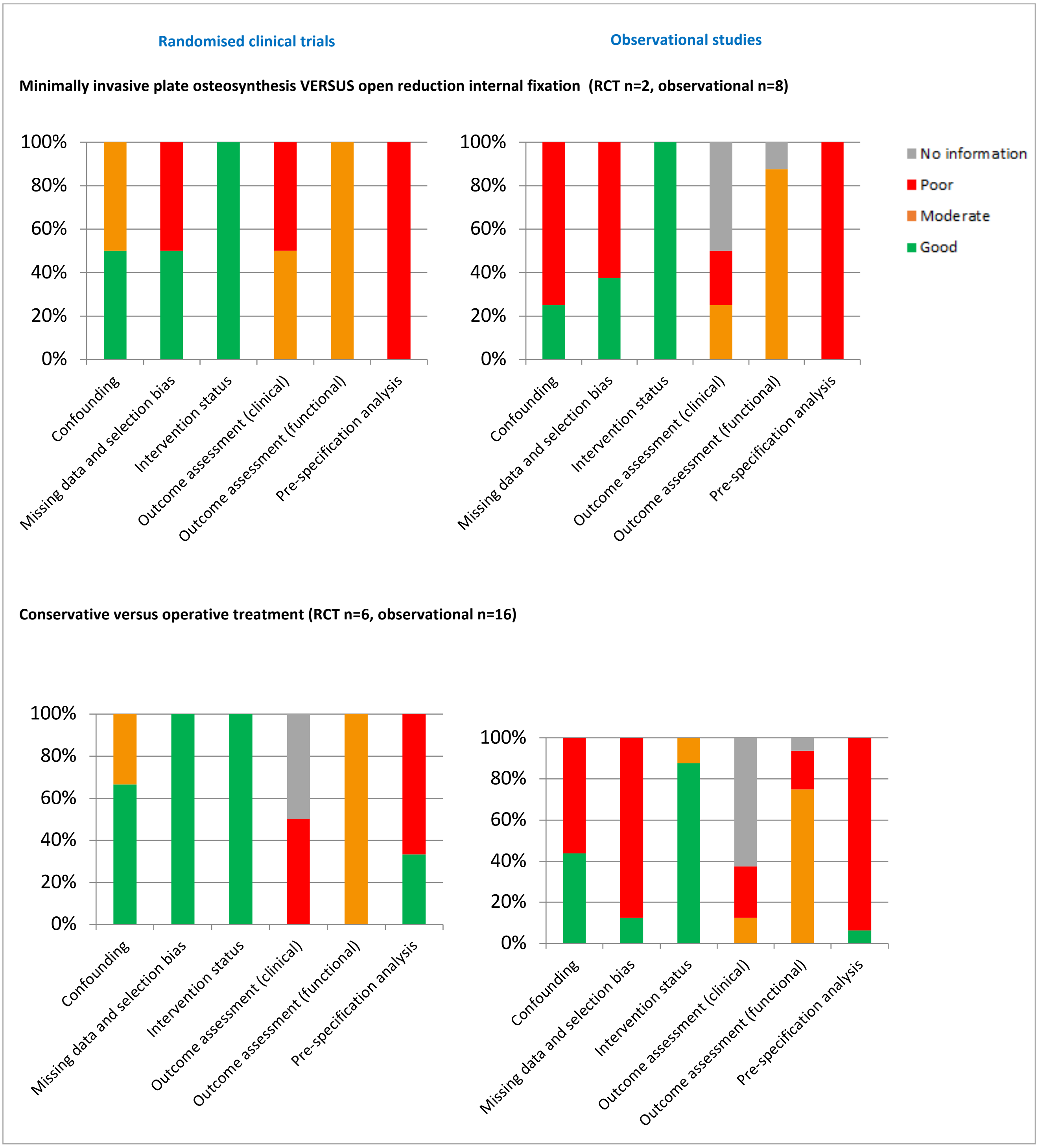

The largest difference between RCTs and observational studies lies in better control for confounding in the former. However, in 3 (42%) RCTs, differences in baseline characteristics between treatment groups were observed. On the other hand, risk of confounding was present in 20 (83%) observational studies. Notably, 9 (37%) observational studies applied methods to control for confounding by using

statistical methods (matching n=3, correction n=2, stratification n=1) or taking precautions in the study design (natural experiments n=3).

The second difference is in the domain "missing data and selection bias". Nineteen (79%) observational studies had missing data in outcome variables and confounder variables. Fifteen of these studies that encountered missing data neither applied methods to address missing data nor gave convincing arguments why their complete case analysis was justified.

Both RCTs and observational studies had low scores in the domain "outcome assessment". This was mostly attributable to lack of a valid and clear definition of the clinical outcome (union or non-union in this example). In 4 (13%) studies, this outcome was clearly defined (using a time-component and clinical/radiological component in the definition). Functional outcomes scored moderately in both study designs. This was due to the combination of using validated functional scores (good methodology) and lack of blinding in the outcome assessment (poor methodology).

Lastly, two (25%) RCTs and one (4%) observational study published a pre-specified protocol prior to the start of the study describing design, data collection, outcome assessment and analysis.

Discussion

This review studied the effects of narrowing or broadening the research question (PICO) in systematic reviews and found that on the applicability of included studies has direct implications for the applicability of studies and with it, the number of studies that may be included in systematic reviews. In addition, we investigated methodological challenges in RCTs and observational studies and found that common methodological issues encountered in both RCTs and observational studies were a lack of clear clinical outcome definitions and pre-specification of study design in a published protocol prior to the start of the study Additionally, while observational studies are typically at higher risk of confounding compared to RCTs, attempts to control for confounding were rarely performed in the assessed observational studies.

With regard to PICO definitions, the results of this study underline two aspects. Firstly, it is not surprising that keeping definitions of the PICO relatively broad allows inclusion of more individual studies in a systematic review.[5] However, it may also lead to larger variation in the patients with the clinical problem of interest (heterogeneity), more variations of the intervention/comparator and to challenges in (diverse) outcome interpretations, as is also shown in the present study.[6] These “broad” PICOs are designed to investigate the clinical benefits and harms of an intervention in a general sense for a large part or even the entire spectrum of the clinical problem. However, systematic reviews with “narrow” PICOs produce results that are, arguably, easier to apply to guide individual patient care.

Secondly, although PICO covers the basic elements (population, intervention/comparator, outcomes) that determine applicability of individual studies, there are other relevant elements that could, but often are not, included in the PICO. For example, the “setting”; If a systematic review aims to investigate a clinical problem in a speciality center (e.g., level 1 trauma center) and incorporated this setting element

in their PICO, then the results of all studies performed in a non-speciality center will be rendered inapplicable. Other examples of elements that further narrow down the PICO and frequently are overlooked include exclusion of patients with comorbidities, intensity and duration of treatment, co-interventions, older treatment modalities not in use anymore, variations in definitions on the same outcome. An extensive list of elements has been described by Atkins et al.[6]

Regarding methodological issues of RCTs and observational studies, the results highlight the following aspects. Control for confounding seems to be an issue in both study designs but have a different cause. Whilst imbalances in baseline characteristics in RCTs most likely stem from a small sample size in the included studies, problems with confounding in observational studies are probably a representation of fundamental differences between treatment groups. Indeed, observational studies inherently are more prone to confounding, but many methods for measured confounding have been proposed, including e.g., propensity score analysis.[7] In the included studies, however, these methods were rarely applied and whether this was intentional could not be checked.

The same phenomenon applies for clear definitions of clinical outcomes. For reasons unknown, the included studies did not give a clear definition of their outcome variables in the methods section despite the existence of clear definitions in current literature for frequently used orthopedic trauma outcomes.[4] This may lead to misclassification of outcomes within studies and introduces heterogeneity in risk estimates between studies, thus hampering the interpretation of pooled analyses in systematic reviews. The lack of clear outcome definitions goes with the lack of pre-specification of study design in a protocol prior to conducting the actual study. Establishing and publishing a protocol, for observational studies just as for RCTs, improves study quality and thus the validity of results.[8]

Several limitations should be mentioned. Firstly, this study only used two systematic reviews as an example for describing effects on PICO component definition and investigate methodological issues in study design. Although this study is meant to increase awareness of these aspects for the entire field of orthopaedic trauma research, it remains uncertain to what degree results can be generalized. Secondly, the appraisal tool used in this study contains a set of applicability and methodological scoring items specifically selected for orthopaedic trauma research. Although it covers the most important discriminative items, it is not all-encompassing covering every possible methodological quality item. Thirdly, the results of this study are based on what was reported in the included reviews/studies, consequently missing out on unreported information.

With regard to defining the PICO, it is important to consider the aim of the systematic review. If a systematic review aims to provide evidence for general treatment guidelines, then it might be advisable to keep definitions of the PICO components broad to allow inclusion of natural variations in study population and interventions reflecting the variations found in the entire spectrum of patients with a clinical problem. If evidence is sought on an individual patient level, it is advisable to consider narrow definitions and include more than just the basic elements into the PICO, such as setting, co-intervention, and treatment intensity/duration.

RCTs in orthopedic trauma research are typically single-center initiatives that are frequently limited by small sample size possibly resulting in imbalances in confounders and a lack of power.[9] Increasing the sample size is not always feasible and observational studies are sometimes considered a reasonable alternative.[10] Often, the sample size is not the limiting factor in an observational study. However, a problem with observational studies in orthopedic trauma appears to be confounding and, as illustrated in the present study, the lack of using control measures, either by design or statistically. We would like to underline the importance of using these control methods in observational studies as it improves the validity of estimates significantly. In case authors decide not to use them, it should be considered to describe the reasons why it is deemed unnecessary.

Another step forward in methodological quality of studies of orthopedic interventions would be explicitly describing clinical outcome definitions in the research manuscript or, preferably, a pre-specified protocol prior to conducting a study.

Conclusion

We conclude that broadening or narrowing down definitions of PICO components (population, intervention/comparator, outcome) has direct consequences for the number of studies that can be included in a systematic review and the level of applicability (individual patient-level or entire spectrum with the clinical problem of interest). Depending on the purpose of the review (treatment decision on patient level or treatment guidelines), definitions of PICO components should be chosen accordingly. Secondly, both RCTs and observational studies in orthopaedic trauma research have recurring problems including the lack of control for confounding and clear outcome definitions. To a large extent these problems could be solved, for example by establishing and publishing a protocol for all studies, irrespective of design (RCTs and observational studies), as it forces researchers to think about critical design aspects of their study.

References

1. K Luijken, B.v.d.W., L Hooft, LPH Leenen, RM Houwert, RHH Groenwold., *How to assess applicability and methodological quality of comparative studies of operative interventions in orthopedic trauma surgery.* unpublished, 2022.
2. Beks, R.B., et al., *Operative versus nonoperative treatment of proximal humeral fractures: a systematic review, meta-analysis, and comparison of observational studies and randomized controlled trials.* J Shoulder Elbow Surg, 2018. **27**(8): p. 1526-1534.
3. Li, F., et al., *Comparison between minimally invasive plate osteosynthesis and open reduction-internal fixation for proximal humeral fractures: a meta-analysis based on 1050 individuals.* BMC Musculoskelet Disord, 2019. **20**(1): p. 550.
4. Paul Pynsent, J.F., Andrew Carr, *Outcome Measures in Orthopaedics and Orthopaedic Trauma.* CRC Press, 2004.
5. Squires, J.E., J.C. Valentine, and J.M. Grimshaw, *Systematic reviews of complex interventions: framing the review question.* J Clin Epidemiol, 2013. **66**(11): p. 1215-22.
6. Atkins, D., et al., *Assessing the Applicability of Studies When Comparing Medical Interventions*, in *Methods Guide for Effectiveness and Comparative Effectiveness Reviews*. 2008: Rockville (MD).
7. Hernán MA, R.J., *Causal Inference: What If.* . Boca Raton: Chapman & Hall/CRC.
8. Kahan, B.C. and V. Jairath, *Outcome pre-specification requires sufficient detail to guard against outcome switching in clinical trials: a case study.* Trials, 2018. **19**(1): p. 265.
9. Investigators, S., et al., *(Sample) size matters! An examination of sample size from the SPRINT trial study to prospectively evaluate reamed intramedullary nails in patients with tibial fractures.* J Orthop Trauma, 2013. **27**(4): p. 183-8.
10. Houwert, R.M., et al., *Study methodology in trauma care: towards question-based study designs.* Eur J Trauma Emerg Surg, 2021. **47**(2): p. 479-484.

Mapping of the signaling questions to item scores

The following tables contain the signaling questions for each item and the mapping algorithms to derive assessment of the overarching item questions. The items are derived based on the Risk of Bias tool-2 (RoB-2) by Cochrane and the Risk Of Bias in Non-randomized Studies - of Interventions (ROBINS-I) tool. The formulation of signaling questions and mapping algorithms in this document stem from the RoB-2 tool and ROBINS-I tool (with slight modifications and additions). We refer to the explanation and elaboration documents of each of these tools for additional information about the background and implementation of the tool, accessible from https://www.riskofbias.info.

Applicability

Item 1: Population

Overarching question

Is the patient population included in the study representative of the patient population defined in the PICO of the systematic review?

Signaling questions

1.1. Did inclusion criteria match the patient population specified in the PICO?

1.2. Was a relevant subgroup of participants excluded?

Mapping

**Signaling question**		**Default applicability assessment for population item**
**1.1. Did inclusion criteria match the patient population specified in the PICO?**	**1.2. Was a relevant subgroup of participants excluded?**	
Y / PY	N / PN	Good applicability
Y / PY	Y / PY / NI	Moderate applicability
N / PN / NI	N / PN	Poor applicability
N / PN / NI	Y / PY / NI	Poor applicability
NI	NI	No information

Item 2: Intervention

Overarching question

Is the investigated intervention representative of the intervention defined in the PICO of the systematic review?

Signaling questions

2.1. Was the investigated intervention similar to the intervention as defined in the PICO?

2.2. Were the participating surgeons experienced in conducting the investigated procedure?

2.3. Was the post-operative treatment regime in the intervention arm similar to the one defined in the PICO?

Mapping

**Signaling question**			**Default applicability assessment for intervention item**
**2.1. Was the investigated intervention similar to the intervention as defined in the PICO?**	**2.2. Were the participating surgeons experienced in conducting the investigated procedure?**	**2.3. Was the post-operative treatment regime in the intervention arm similar to the one defined in the PICO?**	
Y / PY	Y / PY	Y / PY	Good applicability
Y / PY	Y / PY	N / PN / NI	Moderate applicability
Y / PY	N / PN / NI	Y / PY	Moderate applicability
Y / PY	N / PN / NI	N / PN / NI	Poor applicability
N / PN / NI	Any score	Any score	Poor applicability
NI	NI	NI	No information

Item 3: Comparator

Overarching question

Is the comparator intervention representative of the comparator defined in the PICO of the systematic review?

Signaling questions

3.1. Was the comparator similar to the comparator as defined in the PICO?

3.2. Were the health care professionals experienced in conducting the comparator procedure?

3.3. Was the post-intervention treatment regime in the comparator arm similar to the one defined in the PICO?

Mapping

**Signaling question**			**Default applicability assessment for comparator item**
**2.1. Was the comparator similar to the comparator as defined in the PICO?**	**2.2. Were the participating surgeons experienced in conducting the comparator procedure?**	**2.3. Was the post-operative treatment regime in the comparator arm similar to the one defined in the PICO?**	
Y / PY	Y / PY	Y / PY	Good applicability
Y / PY	Y / PY	N / PN / NI	Moderate applicability
Y / PY	N / PN / NI	Y / PY	Moderate applicability
Y / PY	N / PN / NI	N / PN / NI	Poor applicability
N / PN / NI	Any score	Any score	Poor applicability
NI	NI	NI	No information

Item 4: Outcome

Overarching question

Is the outcome representative of the outcome defined in the PICO of the systematic review?

Signaling questions

4.1. Was the outcome measurement similar to the outcome as defined in the PICO?

4.2. Was the timing of the outcome measurement described and similar to the specification in the PICO?

Mapping

**Signaling question**		**Default applicability assessment for outcome item**
**4.1. Was the outcome measurement similar to the outcome as defined in the PICO?**	**4.2. Was the timing of the outcome measurement described and similar to the specification in the PICO?**	
Y / PY	N / PN	Good applicability
Y / PY	Y / PY / NI	Moderate applicability
N / PN / NI	N / PN	Poor applicability
N / PN / NI	Y / PY / NI	Poor applicability
NI	NI	No information

Methodology

Item 5: Confounding

Overarching question

Is there comparability of treatment groups, or are appropriate methods applied to correct for incomparability?

Signaling questions

5.1. RCT: Was the allocation sequence random?

5.2. RCT: Was the allocation sequence concealed until participants were enrolled and assigned to interventions?

5.3. RCT: Did baseline differences between intervention groups suggest a problem with the randomization process?

5.4. Obs: Is there potential for confounding of the effect of the intervention in this study?

5.5. Obs: Did the authors use an appropriate analysis method that controlled for all the important confounders?

5.6. Obs: If 5.5. = Y or PY, were confounders that were controlled for measured adequately?

Mapping

RCTs

**Signaling question**			**Default methodology assessment for confounding item**
**5.1. Was the allocation sequence random?**	**5.2. Was the allocation sequence concealed until participants were enrolled and assigned to interventions?**	**5.3. Did baseline differences between intervention groups suggest a problem with the randomization process?**	
Y / PY	Y / PY	N / PN	Good methodology
Y / PY	Y / PY	Y / PY / NI	Moderate methodology
Y / PY	N / PN / NI	N / PN	Moderate methodology
Y / PY	N / PN / NI	Y / PY / NI	Poor methodology
N / PN / NI	Any response	Any response	Poor methodology
NI	NI	NI	No information

Observational studies

**Signaling question**			**Default methodology assessment for confounding item**
**5.4. Is there potential for confounding of the effect of the intervention in this study?**	**5.5. Did the authors use an appropriate analysis method that controlled for all the important confounders?**	**5.6. If 5.5. = Y or PY, were confounders that were controlled for measured adequately?**	
N / PN / NI	Any response	Any response	Good methodology
Y / PY	Y / PY	Y / PY	Good methodology
Y / PY	Y / PY	N / PN / NI	Moderate methodology
Y / PY	N / PN / NI	Any response	Poor methodology
NI	NI	NI	No information

Item 6: Missing data and selection bias

Overarching question

Were the patients included in the analysis representative of all patients included in the study and was the impact of missing data negligible?

Signaling questions

6.1. Were outcome data available for all, or nearly all, participants?

6.2. Obs: Were exposure data available for all, or nearly all, participants?

6.3. Obs: Were confounder data available for all, or nearly all, participants?

6.4. If 6.1/6.2/6.3 = N or PN: were convincing arguments given for complete case analysis or were methods applied to address missing data?

6.5. Was selection of participants into the study (or into the analysis) based on variables measured after the start of the intervention?

6.6. Do start of follow-up and start of intervention coincide all, or nearly all, participants?

Mapping (see next page)

Mapping

**Signaling question**						**Default methodology assessment for missing data and selection bias item**
**6.1. Were outcome data available for all, or nearly all, participants?**	**6.2. Obs: Were exposure data available for all, or nearly all, participants?**	**6.3. Obs: Were confounder data available for all, or nearly all, participants?**	**6.4. If 6.1/6.2/6.3 = N or PN: were convincing arguments given for complete case analysis or were methods applied to address missing data?**	**6.5. Was selection of participants into the study (or into the analysis) based on variables measured after the start of the intervention?**	**6.6. Do start of follow-up and start of intervention coincide for all, or nearly all, participants?**	
Y / PY	Y / PY	Y / PY		N / PN	Y / PY	Good methodology
Any response	Any response	Any response	Y / PY	N / PN	Y / PY	Good methodology
Any response	Any response	Any response	Y / PY	Y / PY / NI	Any response	Moderate methodology
Any response	Any response	Any response	Y / PY	N / PN	N / PN / NI	Moderate methodology
Any response	Any response	Any response	N / PN / NI	Any response	Any response	Poor methodology
NI	NI	NI	NI	NI	NI	No information

Item 7: Intervention status

Overarching question

Was intervention status correctly classified?

Signaling questions

7.1. Did the recorded intervention status correspond to the intervention actually received?

7.2. Was there cross-over between interventions or non-adherence to the assigned intervention regimen that could have affected participants' outcomes?

7.3. If 7.2. = Y or PY, was an appropriate analysis used to estimate the effect of starting and adhering to the intervention?

Mapping

**Signaling question**			**Default methodology assessment for intervention status item**
**7.1. Did the recorded intervention status correspond to the intervention actually received?**	**7.2. Was there cross-over between interventions or non-adherence to the assigned intervention regimen that could have affected participants' outcomes?**	**7.3. If 7.2. = Y or PY, was an appropriate analysis used to estimate the effect of starting and adhering to the intervention?**	
Y / PY	N / PN		Good methodology
Y / PY	Any response	Y / PY	Good methodology
Any response	Y / PY	Y / PY	Moderate methodology
Any response	Any response	N / PN / NI	Poor methodology
NI	NI	NI	No information

Item 8: Outcome assessment

Overarching question

Was the outcome correctly measured?

Signaling questions

8.1. Was the outcome measurement a valid and reliable measurement of the outcome?
8.2. Were outcome assessors aware of the intervention received by study participants?
8.3. Were the methods of outcome assessment comparable across intervention groups?

Mapping

**Signaling question**			**Default methodology assessment for confounding item**
**8.1. Was the outcome measurement a valid and reliable measurement of the outcome?**	**8.2. Were outcome assessors aware of the intervention received by study participants?**	**8.3. Were the methods of outcome assessment comparable across intervention groups?**	
Y / PY	N / PN	Y / PY	Good methodology
Y / PY	N / PN	N / PN / NI	Moderate methodology
Y / PY	Y / PY / NI	Y / PY	Moderate methodology
N / PN	Any response	Any response	Poor methodology
NI	NI	NI	No information

Item 9: Pre-specification of analysis

Overarching question

Were analyses prespecified and did the study adhere to the specified analysis plan?

Signaling questions

9.1. Was the analysis pre-specified, e.g., in a protocol?

9.2. Are the reported results likely to be a selection of results of multiple analyses?

Mapping

**Signaling question**		**Default methodology assessment for confounding item**
**9.1. Was the analysis pre-specified, e.g., in a protocol?**	**9.2. Are the reported results likely to be a selection of results of multiple analyses?**	
Y / PY	N / PN	Good methodology
Y / PY	Y / PY / NI	Moderate methodology
N / PN / NI	Y / PY / NI	Poor methodology
NI	NI	No information
